# Cytogenetic Abnormalities in Myelodysplastic Syndromes: An Overview

**Published:** 2017-07-01

**Authors:** Mohammad Faizan Zahid, Umair Arshad Malik, Momena Sohail, Irfan Nazir Hassan, Sara Ali, Muhammad Hamza Saad Shaukat

**Affiliations:** Medical Graduate, Aga Khan University, Karachi, Pakistan

**Keywords:** Cytogenetics, Karyotype, Myelodysplastic syndromes, Myelodysplasia, Chromosomal abnormalities

## Abstract

Karyotype is one of the main constituents of the International Prognostic Scoring System (IPSS) and revised-IPSS that are the cornerstones for the prognostication of patients with myelodysplastic syndromes (MDS). Del(5q), –7/del(7q), +8 and –Y are among the most extensively studied cytogenetic abnormalities in MDS. The same applies for normal karyotype. There are hundreds of other rare cytogenetic abnormalities that have been reported in MDS, included but not limited to –X, 3q abnormalities, +13/del(13q), i(17q), +21/–21. However, due to a very low number of patients, their impact on the prognosis of MDS is limited. Knowledge of the molecular consequences of different cytogenetic abnormalities allows us to modify treatment regimens based on drugs most active against the specific karyotype present, allowing for the opportunity to individualize MDS treatment and improve patient care and prognosis.

## Introduction

 Myelodysplastic syndromes (MDS) are a group of heterogeneous hematopoietic stem cell disorders characterized by ineffective hematopoiesis, bone marrow dysplasia and peripheral cytopenias with an increased susceptibility in transformation to acute myeloid leukemia (AML) ^1^^,^^2^ .The rapidly evolving methods in molecular oncology and cellular biology have provided insight into the molecular pathogenesis of MDS, offering great advances in diagnosis, gauging patient prognosis and treatment response^   1 ^.With modern developments in diagnostic techniques, genetic abnormalities such as point mutations and copy-number abnormalities can be detected in a large number of MDS patients^   3 ^.Metaphase cytogenetics are capable of identifying chromosomal abnormalities in up to 50% of patients, though most abnormalities (up to 80%) are detected via single-nucleotide polymorphism (SNP) microarrays and/or array comparative genomic hybridization (CGH) analysis nowadays ^3^^-^^5^ .

More than 50% cases of MDS exhibit somatic point mutations that disrupt vital cellular processes, including but not limited to DNA repair mechanisms, signaling cascades, mRNA splicing and epigenetic gene regulation ^2^^,^^6^ .These data have expanded our understanding of MDS pathogenesis, unravelling biological pathways that can be targeted with novel agents and providing new developments in the treatment of MDS. Different combinations of chromosomal abnormalities and somatic point mutations contribute to the large clinico-pathologic spectrum of MDS^   7 ^.

Disease karyotype contributes to the International Prognostic Scoring System (IPSS) score used in the prognostication of MDS patients. Acquired cytogenetic abnormalities are found in 40-50% of cases with MDS and the clinical implications of each individual karyotype play a crucial role in disease course and management^3^^, ^^5^.

Identification of the specific genes affected by each cytogenetic abnormality has been challenging and the consequences of each abnormality are still being elucidated^7^.Some of the common, as well as rare cytogenetic abnormalities reported in MDS are discussed here.

Del(5q) 

Deletions of the long arm of chromosome 5 (5q) are the most frequently found chromosomal abnormalities in MDS (up to 15% of diagnosed cases) ^3^^,^^8^ . MDS with del(5q) exhibits a heterogeneous clinical picture, divided into two large classifications based of clinico-pathology features, responsiveness to therapy and patient prognosis^   9 ^.One subtype arises after prior exposure to cytotoxic chemotherapy (mainly alkylating agents) and/or radiation exposure and often shows additional chromosomal abnormalities and TP53 mutations ^10^^-^^12^ .This subtype shows increased likelihood of leukemic transformation and shorter overall survival (ranging between 6-17 months, depending on the number/severity of other abnormalities)^       13 ^.Conversely, patients with isolated del(5q) have a relatively better prognosis and a reduced risk of progression to AML (5–16% vs. 30–45%)^   14 ^.Patients with 5q– syndrome are included in this subtype. Patients with multiple cytogenetic abnormalities understandably follow an aggressive disease course with substantially lower complete response (CR) rates to lenalidomide in comparison to those patients with isolated del(5q) (approximately 3% vs. 67%)^   15 ^.Importantly, specific deletions in 5q chromosome also dictate prognosis. For example, one study by Jerez et al.^   16 ^ demonstrated that deletions involving the centromeric and extreme telomeric regions of the 5q chromosome and/or specific genes (such as MAML1andNPM1) are more likely to have additional chromosomal lesions and aggressive disease course. 

More studies have identified additional genes affected by del(5q) and their contribution to the complex pathophysiology of MDS. Loss-of-function mutations in ribosomal protein S14 (RPS14 gene) and several other genes encoding ribosomal proteins are implicated in del(5q) MDS and responsible for the characteristic erythroid phenotype of 5q– syndrome^   17 ^. The haplo-insufficiency of these ribosomal proteins results in impairment of pre-rRNA processing, ribosome synthesis and selective induction of the p53 pathway in erythroid progenitors, halting cell-cycle progression and arresting erythropoiesis^   18 ^. Pre-clinical studies show that inactivation of p53 abrogates the cell-cycle arrest in the erythroid progenitors, confirming the role of aberrant p53 induction in ineffective erythropoiesis in 5q– syndrome^   19 ^. Dysregulation of microRNA (miRNA) has also been identified. miRNA-145 and miRNA-146a in particular are localized to chromosome 5q and are not expressed in del(5q) MDS progenitors. A pre-clinical study by Starczynowski et al.^   20 ^identified that depletion of miRNA-145 and miRNA-146a leads to upregulation of their targets, identified Toll–interleukin-1 receptor domain–containing adaptor protein (TIRAP) and tumor necrosis factor receptor–associated factor-6 (TRAF6), resulting in inappropriate activation of innate immune system pathways and signaling^   20 ^. These events lead to megakaryocytic dysplasia, thrombocytosis, and neutropenia^   20 ^. Another study discussed the role of loss of APC gene, also located on chromosome 5q, in the pathogenesis of MDS in 5q– syndrome^   21 ^.

Lenalidomide is the FDA approved standard of care for low-risk MDS with del(5q)^   22 ^,capable of inducing cytogenetic CR in 50-60% of patients with up to 70% achieving transfusion independence ^23^^,^^24^ . However, in some patients, a fraction of the del(5q) MDS clone remains unaffected by lenalidomide and persists despite CR, foreshadowing eventual disease progression and relapse^   25 ^. In addition, the subgroup of patients harboring TP53 mutations with del(5q) show relative resistance to lenalidomide and are associated with short-lived treatment response ^11^^,^^12^ .These observations highlight the importance of consolidating induction therapy as early as possible after getting the patient in CR.

Monosomy 7, del(7q)

Chromosome 7 anomalies (mainly monosomy 7 or deletion of 7q) are reported in approximately 10% cases of de novo MDS and up to 50% of therapy-related MDS ^3^^,^^26^ . Chromosome 7 abnormalities correlated with worse prognosis and reduced overall survival in MDS and other myeloid malignancies such as AML^   5 ^. Commonly deleted regions on 7q identified in MDS are located at positions 7q22, 7q32-33, and 7q35-36^   27 ^. A recent study by McNerney et al.^   28 ^ demonstrated that the CUX1 gene (encoding a homeodomain protein) is under-expressed in myeloid neoplasms with –7/del(7q). Another recent study analyzing driver mutations in MDS pathophysiology showed that 3.5% of patients harbored inactivating mutations of the CUX1 gene^   29 ^.CUX1 is thought to function as a tumor suppressor gene in myeloid progenitor cells by regulating the expression of proteins governing the cell-cycle ^28^^, ^^29^ .

The MLL5 gene, encoding a histone methyltransferase, is another gene mapped to 7q22. In the murine model, homozygous mutations in MLL5 results in impaired neutrophil function and erythropoiesis and a decreased repopulating capacity of hematopoietic progenitors, even in the presence of self-renewal stimuli^   30 ^.Not only this, but cells with mutated MLL5 alleles showed marked sensitivity to demethylation-induced hematopoietic differentiation^   30 ^. These data indicate that MLL5 plays an important role in myeloid differentiation (via DNA methylation) and warrants its investigation as a predictor of response to hypomethylating agents such as azacitidine in patients with MDS. It is of note that although mutations in MLL5 have not been found in myeloid neoplasia, reduced expression of MLL5 does correlate with poor prognosis in AML^   2 ^. EZH2 is another chromatin remodeler located on 7q36 and is mutated in approximately 6% of MDS cases, correlating with poor prognosis^   31 ^. However, deletions in 7q do not result in the loss of the EZH2 gene^   32 ^.Chromosome 7q deletions are usually quite large and haplo-insufficiency of multiple genes located in the deleted regions contribute to MDS pathology^   7 ^.

Trisomy 8

Trisomy 8 is also a common cytogenetic abnormality. Isolated trisomy 8 occurs in approximately 5% of patients with MDS and correlates with an intermediate prognostic risk (*median overall survival*of*23 months*)^   33 ^. One hallmark of +8 MDS is that the chromosomal aberration is thought to occur late during disease pathogenesis, as evidenced by its detection in myeloid progenitors and near absence in CD34+ stem cells^   34 ^.+8 MDS cells express high levels of anti-apoptotic proteins (such as survivin) and exhibit strong resistance to apoptotic stimuli (such as gamma ray irradiation or withdrawal of growth factors)^   35 ^. Knockdown of these anti-apoptotic proteins abolishes the survival advantage of the +8 MDS clone and represents a potential targeted therapy that can be used in this subgroup of patients^   35 ^. In addition, +8 MDS patients show remarkable response rates to immunosuppressive therapies (up to 67%), indicating an underlying autoimmune pathophysiology associated specifically with trisomy 8^   36 ^.The overexpression of anti-apoptotic proteins confers a survival advantage to cells harboring the +8 karyotype over normal hematopoietic progenitors, allowing the MDS clone to survive the autoimmune microenvironment while normal cells are destroyed as the MDS phenotype develops.

**Sex-chromosome abnormalities (–Y, –X)**

Acquired loss of a sex-chromosome (–Y, in males, –X in females) is an age-related phenomenon, but can also occur in association with hematological malignancies^   37 ^.

MDS patients with isolated loss of the Y chromosome are classified under the ‘very good’ prognosis group^   5 ^. Ever since its first discovery in the 1960s, deletion of the Y chromosome and its relationship with myeloid disorders has been under scrutiny. Since loss of the Y chromosome has been attributed to the normal aging process^   38 ^ and the fact that MDS incidence increases with age, the association between –Y and MDS is unclear^   39 ^. Nonetheless, –Y is suggested to be a potential driver in myeloid disorders as evidenced by the pretreatment predominance of the 45, X, –Y karyotype followed by reappearance of normal karyotype during remission from acute leukemia.^   40 ^ Isolated loss of the Y chromosome is a frequent cytogenetic finding in MDS^   37 ^. A study in 2008 revealed that 14 of 142 patients (9.9%) with loss of chromosome Y developed MDS and reported a 3.8-fold increase in the risk of developing MDS with –Y^   39 ^.Trisomy 15 may also occur concurrently with –Y, however, in the presence of trisomy 15, –Y appears to be benign^   39 ^. Further studies will help elucidate the implications of simultaneous +15 and –Y.

On the other hand, loss of the X chromosome in female patients is a relatively rarer defect (isolated –X: 0.2-0.3% patients; –X in combination with other chromosomal abnormalities: up to 1.5% patients) and correlates with an intermediate prognosis (approximately with a median overall survival of 16 months) ^5^^,^^41^^,^^42^ .Turner’s syndrome is defined by the constitutional loss of the X chromosome, however, patients with Turner’s syndrome do not seem to have an increased risk of developing MDS and other hematologic malignancies than the general population^   43 ^. Although –X is an easily detectable finding on conventional cytogenetics, further analysis such as phytohaemagglutinin-stimulated lymphocytes are required to distinguish constitutional loss of the X chromosome from an acquired loss of the X chromosome restricted to hematopoietic progenitors.^44^

**3q abnormalities**

3q abnormalities such as deletions, translocations and inversionsare rare occurrences in MDS but are categorized as poor-risk features due to short overall survival (median 20 months) ^3^^,^^5^ .Inv(3q) and t(3;3)(q21;q26) usually affect the MECOM gene at the 3q26 locus, leading to abnormal overexpression of the EVI1 (a zinc-finger nuclear protein) which results in uncontrolled proliferation and impaired differentiation of hematopoietic progenitors^   45 ^. Chromosomal instability and interference with the activity of transcription factors (such as PU.1, GATA1 and RUNX1) are apparent mechanisms by which EVI1 overexpression leads to the MDS phenotype ^46^^-^^48^ .

A recent multicenter study highlighted the potential of azacitidine as a specific therapy for MDS patients with 3q lesions. With an overall response rate of 50% (CR: 29%) and a median overall survival of 10.6 months, subgroup analysis also revealed that MDS patients with 3q21 translocations had substantially better response rates and overall survival^     49 ^. In the same study, patients with increased expression of EVI1 without chromosome 3q lesions showed comparable response to azacitidine^     49 ^. Specific therapy of MDS harboring 3q abnormalities can be elucidated with further studies.

**Trisomy 13, Del(13q)**

Trisomy 13 is also an uncommon anomaly in MDS, observed in about 0.2% of patients ^3^^,^^50^ ,but is a recurrent abnormality with increased occurrence in AML (1-2% of cases)^   37 ^. +13 usually presents with advanced MDS with excess of blasts and moderate to severe pancytopenia ^37^^, ^^50^ ,hence categorized as a poor-risk cytogenetic feature (approximate median overall survival of 9.5 months)^   51 ^.+13 shows a propensity for older patients (usually >70 years of age) and male predominance ^37^^,^^50^ .Since +13 is very rare in MDS, most information regarding its clinical implications and effects on prognosis is derived from its appearance in AML patients. Trisomy 13 has shown a strong correlation between abnormalities in the RUNX1 and FLT3 genes. Up to 87.5% of AML patients exhibiting the +13 karyotype show cooperating mutations in the RUNX1 gene, a transcription factor playing a vital role in differentiation of hematopoietic progenitors into mature blood cells^   52 ^. Not only this, but +13 and cooperating RUNX1 mutations are strongly associated with abnormally high expression of FLT3 (up to 5-fold increased expression)^   53 ^. In AML with normal cytogenetics, FLT3 mutations are associated with aggressive disease and poor prognosis^   54 ^.

A report of two cases of AML harboring trisomy 13 showed that single-agent therapy with high-dose lenalidomide was able to induce significant response with durable cytogenetic and morphologic CR^   55 ^.Lenalidomide has already established a prominent role in the treatment of del(5q) MDS and may prove to be an attractive choice for +13 myeloid neoplasms (including MDS), which are usually resistant to standard chemotherapy and hypomethylating agents^   55 ^.Keeping in mind the overexpression of FLT3 in these patients, FLT3 inhibitors may also play a role as ‘individualized’ therapy for +13 MDS^   56 ^, though this needs to be confirmed in clinical studies before its clinical application.

Del(13q) not only occurs in a variety of hematologic malignancies, mainly those of lymphoid cells (chronic lymphocytic leukemia, multiple myeloma) but also occurs in myeloid disorders^   37 ^. In contrast to trisomy 13, deletion of chromosome 3q occurs in about 2% of MDS cases^   57 ^.The RB1 gene, a tumor-suppressor gene involved in cell-cycle control and cellular differentiation, is located in the deleted regions of cells with del(13q)^   58 ^.There is a strong association between the occurrence of del(13q) and therapy-related MDS and therapy-related AML, foreshadowing poor outcomes in this patient subgroup ^57^^, ^^59^ .

**Trisomy 21, monosomy 21**

Constitutional trisomy 21 is very well-known in the context of Down’s syndrome and is associated with an increased risk of AML and acute lymphoblastic leukemia^   37 ^.Besides being a hereditary disease, +21 may also occur as a clonal cytogenetic abnormality in hematologic malignancies. Like +13 (discussed above), +21 occurs much more frequently in AML than in MDS^   60 ^.+21 occurs between 0.3-0.8% of MDS cases and is classified as one of the rare cytogenetic abnormalities in the disease ^5^^,^^61^ . MDS patients with trisomy 21 classically show low absolute neutrophil counts with mild anemia and thrombocytopenia^5^. There may also be an association with chronic myelomonocytic leukemia.^61^The molecular events that arise as a result of +21 are yet to be defined and this represents an area of great interest for future research. Currently, patients with isolated +21 are classified in the intermediate-risk group,^   37 ^ though some studies^   61 ^ have reported that it could be better fitted in the poor-risk group. Prospective studies with large patient numbers will help classify the risk magnitude and prognosis of these patients.

Monosomy 21 is also a rare cytogenetic finding in MDS (isolated –21 in 0.3% cases and in combination with other abnormalities in 0.5% cases) ^3^^,^^5^ . Like trisomy 21, the impact of isolated monosomy 21 on the prognosis of patients is limited due to insufficient number of patients and studies and is currently categorized under the intermediate-risk group^   62 ^. It should be noted that monosomy 21 on karyotype may be present as a technical artifact, occurring randomly while cells are prepared for chromosomal band analysis. Thus, the finding of –21 on karyotype may not represent a true monosomy and should be confirmed with additional techniques such as fluorescence in situ hybridization to avoid false positives^   63 ^.

**Isochromosome 17q**

The i(17q) abnormality is classified under the intermediate-risk category, occurring in about 1% MDS cases as the sole abnormality ^64^^-^^65^. MDS with i(17q) has specific phenotypic features such as profound anemia increased peripheral blood leukocytes showing neutrophils with pseudo-Pelger–Huët anomaly and hyperplastic bone marrow with micromegakaryocytic predominance^64^^-^^65^. The TP53 gene is located on 17p13.1 and while one of the alleles is lost with the i(17q) abnormality,^   65 ^ the absence of mutations in the remaining allele suggests that the loss of other genes on chromosome 17p may play a role in the unique pathogenesis of i(17q) hematologic malignancies^64^. To further corroborate this hypothesis, Fioretos et al.^   66 ^ reported no associations between the i(17q) and TP53 mutations. Recently, myeloid disorders (including MDS) with i(17q) have been proposed to be a distinct clinical entity, one with aggressive disease biology, a higher likelihood of evolution to AML and hence poor outcomes^64^.

**Del(20q), del(12p), del(11q)**

Patients with isolated del(12p) and del(20q) are grouped under ‘good’-risk category based on the cytogenetic classification. The outlook of these patients is relatively favorable. Meanwhile, the median overall survival was 6-9 years in patients with isolated del (12p) and median survival in those with isolated del (20q) was 5-6 years ^3^^, ^^8^^, ^^67^ . Patients with isolated del(11q) have the most favorable prognosis ^3^^,^^5^ . Although, del(20q) has been extensively studied in the context of MDS, none of the genes lost in the deleted regions of 20q have shown association with the development of MDS, indicating that an in-depth study of this cytogenetic abnormalities is warranted to ascertain its role in MDS pathogenesis ^3^^,^^5^ . Loss of the L3MBTL gene (which encodes one of the polycomb-group proteins) in del(20q) is thought to lead to genomic instability, but the L3MBTL gene may not be mutated in MDS^   68 ^. The *ASXL1 *gene regulates epigenetic markers and gene expression by interacting with polycomb-complex proteins, various transcription activators and repressors. It is located at the 20q11 locus, but falls outside the deleted regions of the chromosome in del(20q). ^2^^, ^^69^ ASXL1 is mutated in 10-20% of MDS cases, and corresponds with early evolution into AML and shorter overall survival.^69^

Abnormalities in the ETV6 and CBL genes, located on 12p13 and 11q23, respectively, are also reported in MDS. ETV6encodes one of the ETS transcription factors,^     70 ^ whereas the product of CBL acts as a negative regulator of activated receptor tyrosine kinases^   71 ^.

## CONCLUSION

 The broad heterogeneity of MDS highlights a large variety of abnormalities that underlie disease pathogenesis. Technological advancements have enabled us to identify several new biological abnormalities in patients with MDS and have provided profound insights into MDS pathophysiology. In some cases, unique genetic and non-genetic aberrations are associated with specific cytogenetic abnormalities and are responsive to specific forms of therapy. Detailed characterization of cytogenetic findings and the genes affected by these anomalies will further improve our knowledge of the cellular events that lead to MDS. With this knowledge, treatment approaches can be individualized for each patient, in part based on the cytogenetic abnormalities harbored by the MDS clone. This will not only yield better response rates but will also reduce the toxicities associated with other therapies that would be otherwise ‘suboptimal’ for a specific cytogenetic feature, thereby improving patient quality of life and the overall prognosis of MDS patients.

The rarer cytogenetic abnormalities have an unclear impact on patient prognosis and are presently categorized as intermediate-risk abnormalities and most are not included in the current IPSS and the revised-IPSS used in the prognostication of patients. Emphasis should be placed on the collection of additional cases of rare cytogenetic abnormalities to expand our knowledge of their impact and allow for large studies to take place.
